# Deep eutectic solvent-ultrasound assisted extraction as a green approach for enhanced extraction of naringenin from *Searsia tripartita* and retained their bioactivities

**DOI:** 10.3389/fnut.2023.1193509

**Published:** 2023-06-19

**Authors:** Ezzouhra El Maaiden, Houda El Kahia, Boubker Nasser, Khadija Moustaid, Nagib Qarah, Hassan Boukcim, Abdelaziz Hirich, Lamfeddal Kouisni, Youssef El Kharrassi

**Affiliations:** ^1^African Sustainable Agriculture Research Institute (ASARI), Mohammed VI Polytechnic University (UM6P), Laayoune, Morocco; ^2^Laboratory of Biochemistry, Neurosciences, Natural Resources and Environment, Hassan I University of Settat, Settat, Morocco; ^3^Laboratory of Applied Chemistry and Environment, Hassan I University of Settat, Settat, Morocco; ^4^Department of Chemistry, Faculty of Education-Zabid, Hodeidah University, Hodeidah, Yemen

**Keywords:** anticholinesterase activity, green extraction, naringenin, natural deep eutectic solvent, response surface methodology, *Searsia tripartita*, skin aging, ultrasonic-assisted extraction

## Abstract

**Background:**

Naringenin (NA) is a natural flavonoid used in the formulation of a wide range of pharmaceutical, fragrance, and cosmetic products. In this research, NA was extracted from *Searsia tripartita* using an environmentally friendly, high efficiency extraction method: an ultrasound-assisted extraction with deep eutectic solvents (UAE-DES).

**Methods:**

Six natural deep eutectic solvent systems were tested. Choline chloride was used as the hydrogen bond acceptor (HBA), and formic acid, ethylene glycol, lactic acid, urea, glycerol, and citric acid were used as hydrogen bond donors (HBD).

**Results:**

Based on the results of single-factor experiments, response surface methodology using a Box-Behnken design was applied to determine the optimal conditions for UAE-DES. According to the results, the optimal NA extraction parameters were as follows: DES-1 consisted of choline chloride (HBA) and formic acid (HBD) in a mole ratio of 2:1, an extraction time of 10 min, an extraction temperature of 50°C, an ultrasonic amplitude of 75 W, and a solid-liquid ratio of 1/60 g/mL. Extracted NA was shown to inhibit the activity of different enzymes *in vitro*, including α-amylase, acetylcholinesterase, butyrylcholinesterase, tyrosinase, elastase, collagenase, and hyaluronidase.

**Conclusion:**

Thus, the UAE-DES technique produced high-efficiency NA extraction while retaining bioactivity, implying broad application potential, and making it worthy of consideration as a high-throughput green extraction method.

## 1. Introduction

*Searsia tripartita* (ST) is widespread throughout North Africa, particularly in the Saharan steppes and other arid and semi-arid regions. It is often used in several conventional medical procedures to treat human illnesses and diseases (1). Flavonoids have been found in ST to be responsible for several biological activities, including anti-inflammatory, antidiabetic, antiulcerogenic, antimalarial, antimicrobial, and anti-tumor effects (2–6). Naringenin (NA) is one of the most significant flavonoids found in ST (5). Hence, using NA from ST in pharmaceuticals and healthcare items may provide an alternate source of NA while expanding the accessibility of ST. NA is often utilized as a natural antioxidant because of its potent capacity to scavenge free radicals, and decrease oxidative stress (7, 8). The full extent of its prospective applications has not been achieved because of the constraints imposed by extraction solvents and methods.

Currently, the extraction of NA from medicinal and aromatic plants (MAPs) has been carried out using conventional organic solvents, including methanol and ethanol (5, 9). The low solubility and insufficient quantity of NA in MAPs restrict the extraction’s effectiveness (10). Moreover, most organic solvents exhibit inherent drawbacks such as high toxicity, extreme volatility, and non-degradability, endangering both human health and the environment (11). Environmentally friendly and sustainable solvents have drawn a lot of interest as a solution to the problem (11). Deep eutectic solvents (DESs) have emerged as potential green solvents owing to their flexibility and qualities such as high biodegradability, low cost, simplicity of manufacture, and insignificant vapor pressure (12–14). Hydrogen bond donors (HBD) and hydrogen bond acceptors (HBA) are the components of DESs (15). To create stable fluid systems with lower melting temperatures and distinctive solubilizing activity, the HBD and HBA may establish intermolecular hydrogen bonds (15). For extracting bioactive components from medicinal plants, DESs are better substitutes for organic solvents because of their exceptional qualities (16). The extraction of active components from MAPs is significantly impacted by extraction procedures in addition to solvents (17). Compared to traditional procedures, the use of advanced extraction techniques and environmentally friendly solvents improves the recovery of bioactive chemicals (17, 18). Since the frequency of the ultrasonic waves is modified by the expansion and contraction of the material to be treated, the ultrasound-assisted extraction (UAE) method induces acoustic cavitation as well as thermal and mechanical consequences (11–21). This facilitates the extraction of bioactive chemicals. The UAE offers an advantage over the classic extraction method due to its short extraction time, minimal extraction solvent need, and high extraction yield (22). A statistical experimental technique for simulating the functions of continuous variables is called response surface methodology (RSM). To be more specific, RSM assesses the numerous variables and how they interact to affect the experimental process, illustrates the functional connection between the variables, and offers the ideal experimental circumstances (23). When there are three or more experimental elements, the Box–Behnken design (BBD) is one of the RSMs that are often used to explain the findings of tests since it takes fewer trials than other approaches (24).

The aim of the present study was to establish a highly efficient method for the extraction of NA from ST using ultrasound-assisted extraction with deep eutectic solvents (UAE-DES). RSM with a BBD was used to figure out the optimal extraction parameters for the UAE-DES process based on the results of single-factor studies. High-performance liquid chromatography was used to analyze the yields of NA. After that, the antioxidant properties of NA extracts were assessed, as well as their suppression of enzymes associated with skin aging (collagenase and elastase), hyperpigmentation (tyrosinase), hyperglycemia (alpha-amylase), inflammatory (hyaluronidase), skin damage (elastase and collagenase) and neurological diseases (acetylcholinesterase and butyrylcholinesterase).

## 2. Materials and methods

### 2.1. Chemicals and reagents

Standard NA, L-tyrosine, azo dye-impregnated collagen, *Clostridium histolyticum* collagenase, pig pancreas elastase, 4-(dimethylamino)benzaldehyde (DMAB), phosphate-buffered saline (PBS), N-succinyl-Ala-Ala-p-nitroanilide (AAAPN), potassium tetraborate tetrahydrate (K2B4O7-4H2O) were bought from Sigma-Aldrich Chemical Co. (St. Louis, MO, United States). The solvents lactic acid, formic acid, were acquired from Merck (Darmstadt, Germany). Reagents containing choline glycerol, Citric acid, Ethylene glycol, urea, α-amylase, 1,1-diphenyl-2-picrylhydrazyl (DPPH), 2,2′-Azinobis-(3-ethylbenzthiazoline-6-sulphonate) (ABTS), 6-hydroxy-2,5,7,8-tetramethylchroman-2-carboxylic acid (Trolox) from Thermo Fisher Scientific (Waltham, MA, United States). All solvents and reagents used throughout the experiment were analytical grades.

### 2.2. Plant material

Using conventional fieldwork and collecting techniques (25, 26), the aerial parts of *Searsia tripartita* (Ucria) Moffett (ST) were collected in Jun 2021 from the area of Laayoune Sakia El Hamra, Morocco. Taxonomists identified and verified the plant, and it was placed in the regional ASARI herbarium, Um6p. In a tray dryer, ST was dried for 72 h (60°C). Before being used, dried materials were ground to a size of 250–500 μm and kept in an airtight container (4°C).

### 2.3. Preparation of DESs

The preparation of DESs was conducted as described by Hernández-Corroto et al. (27). The heating technique was used to create six distinct forms of DES, which were then employed as a solvent to extract NA from ST. The HBA and HBD components of DES are explained in Table 1, along with the molar ratio selected according to the optimal conditions found in the literature for the extraction of flavonoids from natural sources. A translucent, colorless liquid was created by heating mixtures in a water bath at 80°C while vigorously shaking them.

**Table 1:** Acceptors of hydrogen bonds (HBAs) and donors of hydrogen bonds (HBDs) used to make deep eutectic solvents (DESs).

Abbreviation	Component 1 (HBA)	Component 2 (HBD)	Mole ratio	References
DES-1	Choline chloride	Formic acid	2:1	(28)
DES-2		Ethylene glycol	1:2	(29)
DES-3		Lactic acid	1:2	(30)
DES-4		Urea	1:2	(30)
DES-5		Glycerol	1:2	(31)
DES-6		Citric acid	2:1	(32)

### 2.4. UAE-DES extraction

An ultrasound device (QSonica Q500, 500 W power, 20 kHz, 25 mm probe, 120 μm maximum amplitude) equipped with a controller of time, temperature, amplitude, and pulse was used for the UAE-DES process. DES and ST powder were thoroughly combined based on the solid–liquid ratio. The mixture was then exposed to sonication at a predetermined amplitude and exposure time. It was then centrifuged for 20 min at 2,600 × *g*, filtered through a paper filter (Whatman No.1, United Kingdom), and condensed in a vacuum rotator.

### 2.5. Single-factor experimental design (SFED)

A single-factor experimental design (SFED) was adopted to identify and select appropriate values from the 5 variables tested: HBA/HBD molar ratio (01,01, 1.5,1, 02,01, 2.5,1, 03:01 and 3.5:1), the water content in DES (20, 30, 40, 50, 60, 70%), the liquid to solid ratio (20, 30, 40, 50, 60, 70 mL/g), the ultrasonic power (30, 60, 75, 90, 115, 130, 150, 180 W), the duration (5, 10, 15, 20, 25, 30, 35, 40, 60, 80, 100 min), and the temperature (30, 40, 50, 60, 70, 80°C). The common conditions selected were as follows: DES-1 molar ratio of 02:01, the water content in DES-1 of 30%; a liquid-to-solid ratio of 30 mL/g; an ultrasonic power of 90 W; an ultrasonic time of 10 min; and an ultrasonic temperature of 50°C. One independent parameter was modified, and the others were held constant. NA was quantified with high-performance liquid chromatography (HPLC) to select the relevant independent parameters that significantly influenced the efficiency of the extraction. Supplementary Table S1 the settings for extracting data from the studies with a single factor are shown. Three duplicates of each experiment were conducted.

### 2.6. Box–Behnken design (BBD) and statistical analysis

Response surface methodology with a BBD was used to figure out the best conditions for the DES-UAE process based on the results of SFED. To generate 46 runs for the response surface test, a 5 variable (X1-X5) was employed. The effective extraction parameters, including ultrasonic time (min), temperature (°C), solid–liquid ratio (g/ml), power (W), and water ratio in DES-1 (%), are listed in Supplementary Table S2 at three levels (−1, 0, and + 1). NA yield was the response variable, and HPLC analysis was used to quantify it. In the analysis of variance (ANOVA), a *p*-value of fewer than 0.05 means that the conditions being studied were significant, and a *p*-value of fewer than 0.0001 means that they were very significant. In addition, if the *p*-value is greater than 0.10, it means that the factor is not significant. The insignificant factors were preliminarily removed.

### 2.7. HPLC analysis

The NA was measured using an Agilent 1200 HPLC system (Agilent Technologies, CA, United States). The samples were separated at 40°C using a Capcell PAK C18 column with an internal diameter of 250 × 4.6 mm and a particle size of 5 μm. The flow rate was 1 mL/min, and the injection volume was 10 μl. Water: formic acid (95.5, v/v), and methanol (100%) made up the mobile phase (A). The binary gradients were: 0–25 min, 20–60% B, 25–25.1 min, 60–100% B, 25.1–30 min, 100–60%B, 30–30.1 min, 60–20% B, and 30–35 min, 20% B. At 280 nm, the absorbance was measured. By comparing the UV–vis spectra and peak retention durations to the original NA reference standard, peaks were identified. Results are reported as (μg/g DW) after two analyses of the samples were performed (8).

### 2.8. Antioxidant and enzymatic inhibition assays

#### 2.8.1. Antioxidant activities

The antioxidant activity was measured using DPPH and FRAP tests. 0.4 mL of the sample solution (0.025–0.2 mg/mL) was added to 2 mL of the DPPH methanolic solution (0.2 mM) for the DPPH radical scavenging experiment, and the combination was incubated in the dark for 30 min before the absorbance was measured at 517 nm. DPPH radical scavenging activity was measured in milligrams of trolox per gram (33). To conduct the FRAP experiment, 0.5 mL of potassium ferricyanide (1%) was combined with 0.2 mL of NA sample (0.025–0.2 mg/mL). The resultant mixture was then incubated for 20 min at 50°C. After adding 2.5 mL of 10% trichloroacetic acid to the mixture, it was centrifuged at 2200 × *g* for 20 min. Combining 0.5 mL of the top layer with 0.1 mL ferric chloride and 0.5 mL of distilled water yielded a solution. FRAP was estimated as mg TE/g material after measuring absorbance at 700 nm (33).

#### 2.8.2. Cholinesterase inhibition assays

According to the literature, acetylcholinesterase (AChE) and butyryl-cholinesterase (BChE) were used to perform *in vitro* anticholinesterase activities (34). Briefly, 50 μL of NA sample solution (0.025–0.2 mg/mL) was coupled to 5,5-Dithio-Bis(2-Nitrobenzoic) acid plus 25 μL of AChE or BChE solutions in microtiter plates with 96 wells and then incubated at 25°C for 15 min. Following the addition of 25 μL of acetyl-thiocholine iodide or butyryl-thiocholine chloride, the solution was incubated for 10 min at 25°C for AChE and BChE, respectively. At 405 nm, the absorbance was measured. The AchE and BchE *in vitro* inhibitory activities were determined in milligrams of galantamine equivalents per gram of material (mg GALAE/g).

#### 2.8.3. Alpha-amylase inhibition assay

α-amylase inhibition activity was evaluated according to McCue and Shetty (35). Briefly, 50 μL of the α-amylase solution produced in phosphate buffer was mixed with 25 μL of the sample solution (0.025–0.2 mg/mL) in 96-well microplates. The reaction began by adding 50 μL of 0.5% starch solution to the microplates, which were then incubated for 10 min at 37°C. At 630 nm, the absorbance was measured after adding 25 μL of 1 M HCL and 100 μL of potassium iodine solution (0.25 mM) (35). The α-amylase inhibitory action was reported in mmol ACE/g.

#### 2.8.4. Tyrosinase inhibition assay

Utilizing L-DOPA as a substrate, the tyrosinase inhibition experiment was carried out in accordance with the technique previously described by Chiari et al. (36). The sample mixture (20 μl, 0.025–0.2 mg/mL), aqueous fungal tyrosinase solution (10 μl, 50 units/mL), and phosphate buffer (pH 6.8, 80 L) were combined and incubated for 5 min at 37°C. Next 90 μl of L-DOPA (2 mg/mL) was added. The mixture was then incubated at 37°C for 20 min. At a wavelength of 475 nm, the concentration of dopachrome was determined. As a control, kojic acid in dimethyl sulfoxide was utilized. The inhibition rate of tyrosinase was computed as follows:


$$\mathrm{Tyrosinase inhibition rate}\left(\%\right)=\left[\begin{array}{l} \left(\mathrm{Acontrol}-\mathrm{Asample}\right)\\ /\mathrm{Asontrol} \end{array}\right)\times 100$$

#### 2.8.5. Collagenase inhibition assay

The Barrantes and Guinea (37) method was applied to determine the inhibiting collagenase activity. After adding 800 μL of 0.1 M Tris–HCl (pH 7) and 100 μL of the sample (0.025–0.2 mg/mL) to each tube, a consistent mass of 1 mg of collagen that had been penetrated with Azo dye was measured in the tubes. The mixture was then immediately mixed with 100 μL of collagenase (200 units/mL), and it was let sit for an hour at 43°C. The tubes were then centrifuged for 10 min at 3000 rpm. Each tube’s supernatant was collected on 96-well plates. Negative control was carried out using buffer and substrate but no enzyme. Fluorescence was measured at 495 nm for excitation and 515 nm for emission. All experiments were carried out in duplicate and independently. The inhibition rate of collagenase was determined using the following formula:


$$\mathrm{Collagenase inhibition activity}\;\left(\%\right)=\left(\begin{array}{l} 1-\mathrm{Sabs}-\mathrm{Sbabs}\\ /\mathrm{Cabs}-\mathrm{Cbabs} \end{array}\right)\times 100$$

Where S is the test sample’s absorbance, Sb is the blank sample’s absorbance, C is the positive control’s absorbance, and Cb is the negative control’s absorbance.

#### 2.8.6. Elastase inhibition assay

Using the porcine pancreatic elastase enzyme inhibition test, the *in vitro* elastase-inhibiting activity was assessed (38). This examination was conducted in a buffer containing 0.2 M Tris–HCL (pH 8.0). To create a 100-unit stock solution of porcine pancreatic elastase, the enzyme was dissolved in sterile water. In the buffer, the substrate N-Succinyl- Ala-Ala-p- nitroanilide (AAAPVN) was dissolved. Before adding the substrate to initiate the reaction, the enzyme was incubated for 25 min with the plant extracts. 250 μL of buffer, 10 μg/mL AAAPVN, 0.001 unit of porcine pancreatic elastase, and 25–200 μg/mL of samples comprised the reaction mixture. Maximum velocities (Vmax) were monitored at 410 nm for 25 min at 30-s intervals, and the inhibition rate was computed by employing the equation:


Elastase inhibition%=[(Acontrol−Asample)/Acontrol]x100

Where Asample is the sample’s absorbance and Acontrol is the assay’s absorbance when the buffer is used in lieu of an inhibitor (sample). Quercetin was the active control drug that was used. The chemical used as a control was tris–HCl.

#### 2.8.7. Hyaluronidase inhibition assay

The inhibition of hyaluronidase was measured as previously reported by Sahasrabudhe and Dedhar (39). 50 μL of the sample (0.025–0.2 mg/mL) was dissolved in 5% dimethyl sulfoxide, followed by the addition of 10 μL of bovine testicular hyaluronidase type 1-S (4200 units/mL) in 0.1 M acetate buffer (pH 3.5). The mixture was then put in a 37°C water bath for 20 min of incubation. Once hyaluronidase activated Ca2+ had been treated with 50 μL of sodium hyaluronate (12 mg/mL) dissolved in 0.1 M acetate buffer (pH 3.5), it was incubated at 37°C for 40 min. The mixture was combined with 10 μL of 0.9 M sodium hydroxide and 20 μl of 0.2 M sodium borate before being incubated in a water bath at boiling temperature for 3 min. After cooling to ambient temperature, the reaction was heated in a 37°C water bath for 10 min. Afterward, 50 μl of a p-dimethylamino benzaldehyde solution was added (0.25 g of p-dimethylamino benzaldehyde dissolved in 21.88 mL of 100% acetic acid and 3.12 mL of 10 N hydrochloric acid). The control group was given 50 μl of 5% dimethyl sulfoxide instead of the sample. At 585 nm, the absorbance was measured. Tannic acid was utilized as a comparative standard. Using the following formula, the enzyme inhibition rate was calculated.


$$\mathrm{Hyaluronidase inhibition}\%=\left[\begin{array}{l} \left(\mathrm{Acontrol}-\mathrm{Asample}\right)\\ /\mathrm{Acontrol} \end{array}\right]\;x\;100$$

The absorbance of the sample extracts is denoted by “Asample,” and the absorbance of the test conducted in the absence of an inhibitor is denoted by “Acontrol.”

### 2.9. Statistical analysis

One-way analysis of variance (ANOVA) and Tukey’s test were used to look at the data from the single-factor experimental design from a statistical point of view. Response surface modeling (RSM) was used with version 7.0.0 of Design Expert (Stat-Ease, United States). This was done for optimization and modeling.

## 3. Results and discussion

### 3.1. Single-factor analysis of NA extraction

#### 3.1.1. Impact of DES’s on NA extraction

**Figure 1:**
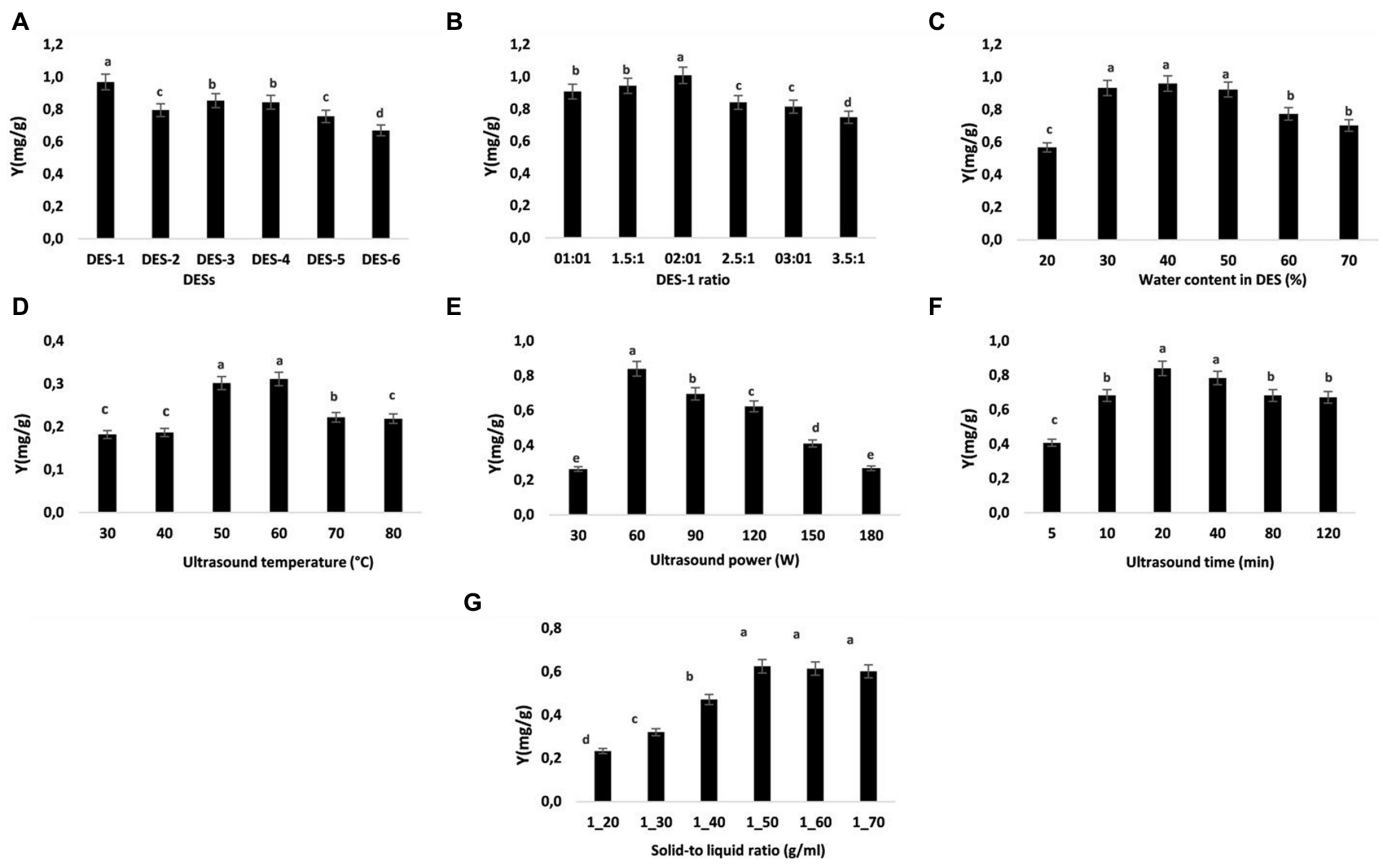
Impact of various extraction variables on NA extraction efficiency. **(A)** DES types, **(B)** DSE-1 molar ratio, **(C)** water content in DES-1, **(D)** extracting temperature, **(E)** ultrasonic power, **(F)** ultrasonic duration **(G)**, and solid–liquid ratio. The data are reported as means ± SD (*n* = 3). A significant difference (*p* < 0.05) is indicated by data with different letters **(A–D)**.

To choose an appropriate DES for NA extraction, six distinct kinds of DES were synthesized based on the various hydrogen bond donors (formic acid, oxalic acid, lactic acid, urea, glycerol, and malic acid). Figure 1A demonstrates how the extraction efficiency changes with various DESs. The highest NA yield was achieved by choline chloride-formic acid (DES-1: 0.97 mg/g). There were significantly lower yields for NA extraction when using choline chloride-citric acid (DES-6: 0.67 mg/g) compared to the other DESs we tested. Because of its greater electrostatic interaction with flavonoids than the other DESs, DES-1 demonstrated better extraction efficiency than the others (40). Moreover, formic acid and choline chloride, which exist naturally in many whole foods, are totally safe to ingest (41). The DES system (DES-1) composed of choline chloride and formic acid was used in the following investigations.

#### 3.1.2. Impact of DES mole ratio on NA extraction

As previously mentioned, the molar ratio of HBD to HBA in the DES regulates the interaction of hydrogen bonding, which in turn influences the viscosity of the DES (42). The physical properties of DES also have an impact on the efficiency with which NA is extracted (41, 42). We did not attempt to increase the choline chloride level since DES crystallizes at a molar ratio greater than 3.5:1. It can be seen in Figure 1B that the selectivity and extraction rate of NA increase with the mole ratio of choline chloride (ChCl) to formic acid (FA), reaching maximum values at a 2:1 mole ratio of ChCl to FA. The increase in ChCl content in DES may increase the interaction of NA with DES, which increases the selectivity and extraction rate. However, when the mole ratio of ChCl to FA is further increased from 2.5:1 to 3.5:1, the selectivity and extraction rate of NA decrease. Due to this, the mole ratio of ChCl-FA in DES-1 used in the subsequent experiments was 2:1.

#### 3.1.3. Impact of water content in DES on NA extractionextraction

As discussed earlier, the molar ratio of HBD to HBA in the DES controls the hydrogen bonding interaction, which affects the viscosity and surface tension of DES (43). As a result, optimizing the mass transfer rate and processing efficiency relies on selecting appropriate water content in DES. Figure 1C demonstrates how the amount of water present in the DES-1 significantly affected the extraction yield. Increasing the water ratio in DES-1 from 20 to 50% led to an important improvement in the extraction yield of NA. The high extraction yield may have been achieved because DES-1 viscosity and polarity were optimized by adding water, resulting in more effective interactions between ST and DES-1 (43, 44). Yet, the extraction efficiency dropped dramatically when the water content was brought up to 50%. The low viscosity and great fluidity of DES-1 with a water content of 30–50% made it less expensive to manufacture. Thus, for the next tests, DES-1 with 30–50% water content was chosen.

#### 3.1.4. Impact of ultrasonic temperature on NA extraction

The successful extraction of bio-compounds from plant material requires optimizing a moderate temperature while keeping economic considerations in mind (45). The optimal temperature for this investigation was between 50 and 70°C. Figure 1D illustrates how yield increased as temperature rose because of enhanced solvation, increased material porosity, and mass transfer. Due to the solvent’s diffusivity in the tissue matrix, the increase in temperature also reduces surface tension and viscosity in extracts, improving extraction yield (46). The quantity of NA that was extracted increased as the temperature rose. The largest quantity of NA that could be extracted was achieved at a temperature of 60°C, after that, when the temperature was raised, the rate of NA extraction fell. This can be mostly since raising the temperature to a certain point enhances NA release. According to this theory, the deterioration of the desirable components occurs at higher temperatures (beyond the optimal temperature) because more cavitation bubbles develop and then collapse, creating shear stress (47). According to a study on the thermal stability of NA (48), the compound is most stable when heated to 60°C. Ultrasonic extraction coupled with thermal hydrolysis significantly improved the extraction of NA from the albedo parts of *Citrus paradisi*, as reported by Stabrauskiene et al. (8), who also found that 50°C provided a better yield of NA than 70°C. As a result, the concentration of NA in the subsequent studies was highest between 50 and 70°C.

#### 3.1.5. Impact of ultrasonic power on NA extraction

Under the established extraction conditions (temperature, time, solid–liquid ratio, and water content in DES-1 were kept constant at 50°C, 10 min, 1/30 g/mL, and 30%, respectively), the effects of different extraction powers (30, 60, 75, 90, 115, 130, 150, and 180 W) on NA yields are presented in Figure 1E. As the extraction power was increased to 60 W, the NA content was enhanced gradually. The extraction efficiency of natural compounds can be clearly increased by enhancing the speed of molecular motion and solvent penetration because of the mechanical effect, acoustic cavitation, and cavitation intensity characteristics (49, 50). Nonetheless, when the power further increased, the extraction yields of NA declined. During the extraction, the increase in power will cause a lot of impurities, which will lead to a decrease in NA yields. Consequently, the ultrasonic power was set between 60 and 120 W in the following investigations.

#### 3.1.6. Impact of ultrasonic time on NA extraction

The period of ultrasonic exposure has a considerable effect on the extraction of compounds (51). Longer extraction times have been recorded during traditional extraction techniques geared toward the hydrolysis and oxidation of phenolic compounds (52). Ultrasound-assisted extraction is acknowledged as a sophisticated approach that may expedite the mass transfer and consequently improve extraction kinetics (53). Consequently, a helpful aspect of this technology is the shortened extraction time with less solvent usage. The optimal extraction would aid in lowering the method’s energy and cost, as well as making commercialization simpler in pharmaceutical and other relevant industries (53). In the current investigation, the extraction of NA from ST was performed at 5–120 min (Figure 1F). The quantity of NA extracted rises with increasing extraction time, reaching a maximum of 20 min (0.84 mg/g) before beginning to fall. Increased NA extraction by increased release of NA into the solvent from the powder may occur with extended exposure, however, this may also result in NA deterioration, according to (51). To save time and energy and improve the extraction of NA, the following studies used extraction times ranging from 10 to 40 min.

#### 3.1.7. Impact of solid–liquid ratio on NA extraction

As the biomass-to-solvent ratio has a substantial effect on NA yield, optimizing this variable is crucial for maximizing extraction (54). A solid–liquid ratio between 20 and 70 mL/g was used to extract NA from ST. The ratio of 1–10 g/mL produced the greatest extraction yield of NA, following which equilibrium was restored (Figure 1G). When the solid/liquid ratio is low, more solvent is available to infiltrate the plant matrix, hence enabling the bioactive compounds to dissolve and boosting the extraction yield (55). Hence, the optimal range for testing is between 1/40 and 1/60 g/mL (solid/liquid).

### 3.2. Optimization of UAE-DES and validation of the model

#### 3.2.1. Statistical analysis

The Design Expert program (version 10.0.7) was used to investigate the key UAE parameters using the RSM according to SFED findings. To maximize the interacting effects of five factors, Supplementary Table S3 displays data from 46 trials with five factors and three levels. The Table 2 presents a summary of the ANOVA findings for Y (NA yield mg/g) as a response. The *p*-value of the regression model, which is 0.0001, demonstrates the model’s high significance. Because of how well the model suited the data, the *p*-value for the lack of fit was just 0.1268, indicating that it was not statistically significant. Very high significance (*p* < 0.0001) was found for the model parameters X1, X2, X5, X1X2, X2^2^, X3^2^, and X5^2^. In other words, X1X4, X2X4, X2X5, and X1^2^ all had statistically significant *p*-values (*p* < 0.05). The remaining factors did not reach statistical significance (*p* > 0.10). Table 2 presents the results of the analysis of variance of the model, which reveals that the most influential factors on NA extraction in ST were ultrasonic temperature (with three significant interaction conditions: X1X2, X2X4, and X2X5), duration of ultrasonication (with two significant interaction conditions: X1X2, X1X4), and DES-1 water content (with one significant interaction condition: X2X5). The connection between ultrasonic power, time, temperature, solid–liquid ratio, and water content in DES-1 is shown as a quadratic equation below:


$$\begin{array}{c} Y=10.38-1.07X1-0.69X2+0.29X5+0.70X1X2\\ +\;\;0.22X1X4-0.18X2X4-0.31X2X5+0.18X1^{²}\\ -\;\;0.57X2^{²}+0.26X3^{²}+0.31X5^{²} \end{array}$$

Where Y is the NA yield in micrograms per gram (μg/g)and X1, X2, X3, X4, and X5 are five numeric variables representing the amount of water in DES-1, the solid–liquid ratio, the time of ultrasonography, and the temperature, respectively. Both the raw and adjusted model correlation coefficients (R^2^) were used to assess the strength of the relationship between the variables in question and the observed response (Y). With an adjusted R^2^ of 0.9618, a strong model fit is shown, and with an R^2^ of 0.9812, a high prediction for the response model is indicated. Both the R^2^ and the modified R^2^ demonstrate a significant relationship between the theoretical and empirical estimates. Also, the corrected R^2^ of 0.9279 is quite like the expected R^2^ of 0.9279 for Y in this scenario (Table 2). For all 46 experiments, the average output of NA was between 885.68 and 1244.66 μg/g (Supplementary Table S3). Trial 1’s conditions of 10 min of ultrasonication at 50°C, 60 milliliters of solvent per gram of solid, and 75 watts of ultrasonic power produced the highest extraction yield for NA (Supplementary Table S3).

**Table 2:** ANOVAs for the regression models.

Source	*F*-value	*P*-value	Significance
Model	65.10	<0.0001	**
X1	624.21	<0.0001	**
X2	272.03	<0.0001	**
X5	75.79	<0.0001	**
X1X2	60.21	<0.0001	**
X1X4	5.26	0.0306	*
X1X5	2.83	0.1049	*
X2X4	8.13	0.0089	*
X2X5	10.27	0.0036	*
X1^2^	9.69	0.0043	*
X2^2^	110.42	<0.0001	**
X3^2^	23.47	<0.0001	**
X4^2^	0.75	0.4054	*
X5^2^	29.21	<0.0001	**
Lack of fit	2.92	0.1268	
*R*^2^	0.9821		
Adjusted *R*^2^	0.9618		
Predicted *R*^2^	0.9279		

* Significant; ** highly significant.

#### 3.2.2. Response surface UAE factor interactions

**Figure 2:**
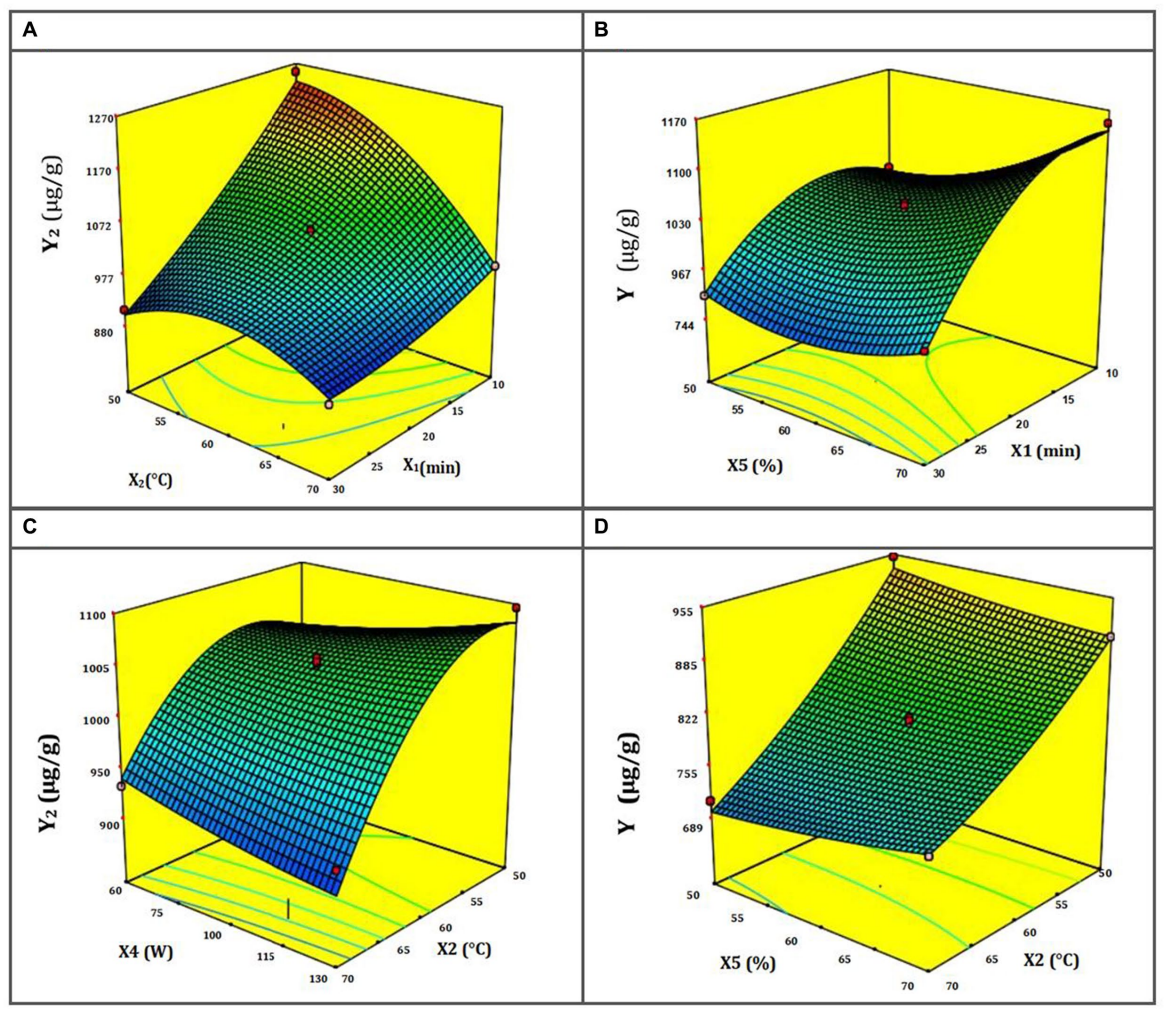
**(A–D)** Response surface for NA DES-UAE from ST as a function of the temperature and duration of ultrasound **(A)**; the power and duration of ultrasound **(B)**; the power and temperature of ultrasound **(C)**; and the water content in DES-1 and the temperature of ultrasound **(D)**.

The response surfaces for NA extraction show the interplay of UAE-DES extraction parameters (Figures 2A–D). Figure 2A shows a response surface depicting the temperature’s dependence on the ultrasound’s exposure time. When the combined value of these two parameters is increased, the rate at which NA is extracted lowers. In addition, the figure reveals that the maximal extraction efficiency of total NA rises at low temperatures but falls at high ones, namely those over 70°C. It’s possible that the extremely high temperatures used in extraction damage the flavonoid structures, leading to this outcome (56). Figure 2B demonstrates that at the maximum ultrasonic duration of 45 min, an increase in ultrasonic power results in a greater extraction yield. Although the greatest quantity of NA extraction yield did decrease with increasing microwave ultrasound time, this was not always the case. This is because ultrasonic waves may reduce the sample’s NA if exposed to them for too long (57). The response surface, shown in Figure 2C, demonstrates how ultrasound time and temperature interact to alter the yield of NA extraction. Hence, the longest duration and the coolest ultrasonic temperatures should provide the largest yield. Nevertheless, raising the temperature to 80°C resulted in a decrease in extraction yield as time was increased. There is a trade-off between improving mass transfer and extraction yield and utilizing high ultrasonic temperature, since the latter may disrupt the structure of flavonoids, which are sensitive to damage (58). Figure 2D shows that a higher NA yield is achieved when the ultrasonic temperature is lowered, and the DES-1 sample’s water content is increased. It has also been shown that, at very high temperatures, extraction yield rises in tandem with water content. This evidences a strong interplay between these two parameters on the response surface during NA extraction.

### 3.3. Antioxidant and enzyme inhibitory activities

Several biological properties have been attributed to NA, including antioxidant, anti-neurodegenerative, anti-diabetic, anti-inflammatory, anti-aging, and others (7). To determine whether the developed UAE-DES method enabled the preservation of the bioactivities of NA, antioxidant activities, and inhibition of key enzymes in diabetes (α-amylase), hyperpigmentation, and skin aging (tyrosinase, elastase, collagenase), inflammation (hyaluronidase), and neurodegenerative disorders (acetyl and butyryl-cholinesterases) were assessed.

**Figure 3:**
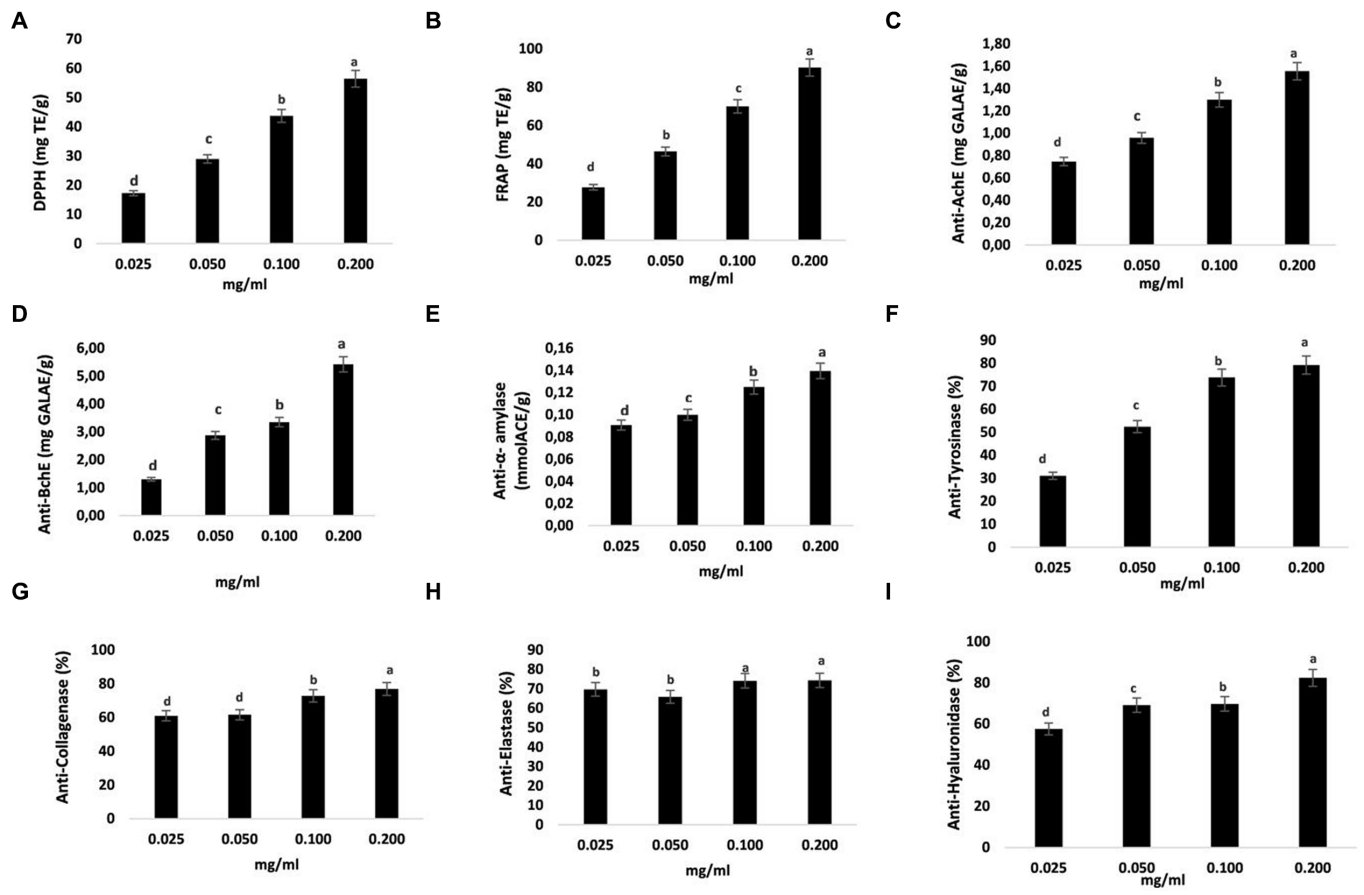
Antioxidant and enzyme-inhibiting abilities of ST samples. Trolox equivalents per gram (mgTE/g) were used to represent DPPH and FRAP **(A,B)**. The inhibitory capabilities of acetylcholinesterase (AchE) and butyrylcholinesterase (BchE) were represented in milligrams of galantamine equivalent per gram (mg GALAE/g) **(C,D)**. The amount of acarbose equivalent per gram of sample (mmol ACE/g extract) was used to indicate the inhibitory ability of α-amylase **(E)**. Tyrosinase inhibition is shown as mg KAE/g extract, meaning milligrams of kojic acid equivalent per gram of sample **(F)**. Collagenase **(G)**, elastase **(H)**, and hyaluronidase **(I)** inhibition capacities are shown as percentages (%). The data are shown as mean SD (*n* = 3). Data with different letters indicate significant differences (*p* < 0.05) **(A–I)**.

Reactive oxygen species (ROS) are produced in excess relative to their ability to be squelched, which results in oxidative stress, which may be prevented by antioxidants. The antioxidant properties of the examined samples (0.025–0.2 mg/g) were evaluated using two assays (DPPH and FRAP). The DPPH assay is a common method for evaluating how well a sample can get rid of 2,2-diphenyl-1-picrylhydrazyl. FRAP activity is based on the samples’ capacity to transform the ferric-tripyridyltriazine complex into a colorful ferrous form. Equivalents of Trolox (TE) were used to get data on metal chelating assays and radical scavenging (Figures 3A,B). The highest activity was found at 0.2 mg/g (56.44 and 90.25 mg TE/g, for DPPH and FRAP assays, respectively). When concentrations rose across the testing range of 0.025–0.2 mg/g, antioxidant activity (DPPH and FRAP) increased as well. Naringenin was shown to be effective in preventing oxidative damage in several studies, including one that found that it protected rats’ liver and kidneys from lead-induced oxidative damage (59) and that it had the capacity to prevent arsenic-induced oxidative liver and renal dysfunction (60).

The neuroprotective effects of naringenin against Alzheimer’s disease (AD)-like neurodegeneration caused by intracerebroventricular streptozotocin are well established (61). Moreover, naringenin protects neurons in a 6-hydroxydopamine (6-OHDA)-generated model of Parkinson’s disease and protects them from 6-OHDA neurotoxicity (62–64). Intriguingly, naringenin ameliorates memory loss caused by type 2 diabetes by enhancing cholinergic function via decreasing hippocampus cholinesterase activity (65). An inhibitor of an enzyme is a substance that binds to an enzyme and inhibits it from performing as intended. The scientific literature has described several inhibitors for a variety of diseases. Based on these results, we attempted to investigate the NA potential for protection as well as its ability to inhibit the enzymes cholinesterase, alpha-amylase, and hyaluronidase. We found that the activities of AchE and BchE within the (0.025–0.2 mg/g) concentration range increased with increasing concentrations, with the highest activity occurring at 0.2 mg/g (1.56 and 5.43 mg GALAE/g) (Figures 3C,D). The activity of α-amylase was clearly suppressed by NA, as shown in Figure 3E, and this inhibition was directly proportional to the concentration of NA. The highest α-amylase inhibitions measured for NA were 0.14 mmol ACE/g at a concentration of 0.2 mg/g. These findings suggested that NA was an effective choice for enzyme inhibitors due to its significant hypoglycemic effects and inhibitory action on α-amylase.

The skin’s structural framework is provided by the extracellular matrix (ECM), which is composed of structural proteins including collagen, elastin, and microfibrils (66). As the skin ages, these factors change, giving the appearance of dry, wrinkled, and loose skin. The melanosome membrane contains tyrosinase, a metalloprotein. This versatile protein catalyzes the first two steps in the synthesis of melanin and converts tyrosine to dopaquinone. Collagen and elastin are key ECM components. One of the primary reasons for intrinsic skin aging is the destruction of these molecules by the enzymes collagenase and elastase, and these substances guarantee the skin’s resilience and elasticity (66). Moreover, during tissue remodeling, hyaluronidase hydrolyzes the ECM, and chronic inflammatory disorders are linked to elevated hyaluronidase activity (67). According to the existing evidence, hyaluronidase inhibitors might be utilized to treat and prevent inflammatory disorders. In this investigation, the inhibitory effects of different doses of the NA extracted using the well-established UAE-DES procedures on tyrosinase; collagenase, elastase, and hyaluronidase were evaluated. In accordance with our findings, NA from ST substantially and dose-dependently suppressed an enzyme related to skin aging. Tyrosinase, collagenase, elastase and hyaluronidase were all significantly suppressed by 0.2 mg/g NA (by 79.2, 76.88, 74.29% and 82.36 respectively) (Figures 3F–I).

## 4. Conclusion

In summary, a sustainable and efficient DES-based UAE extraction method was used for the first time for the optimal recovery of NA from ST, enabling the preservation of its bioactivities. Based on the results of the single-factor experiment, BBD and RSM were utilized to identify the main parameters and optimize the extraction conditions. Out of the six DES investigated in this study, DES-1 (ChCl-FA) had the highest extraction efficiency for recovering NA from ST. A confirmation experiment (*n* = 3) was performed using optimized independent extraction variables (DES-1 consisted of choline chloride (HBA) and formic acid (HBD) in a mole ratio of 2:1, an extraction time of 10 min, an extraction temperature of 50°C, an ultrasonic amplitude of 75 W, and a solid–liquid ratio of 1/60 g/mL), and the experimental yields were compared with predicted values for model validation. With these optimized conditions, the predicted response for NA yield was approximately 1.154 mg/g, and the experimental value was found to be 1.244 mg/g. As a result, the model was verified because the experimental values were found to be near the predicted values. The results of bioactivities indicated that the extracted NA exhibited excellent antioxidant, alpha-amylase, tyrosinase, elastase, collagenase, hyaluronidase, acetyl, and butyryl-cholinesterase inhibition activities. The verification studies under idealized settings demonstrated the method’s dependability and preservation of NA bioactivities. This study lays a strong scientific basis for future medicinal and cosmetic applications as well as a viable, environmentally friendly, and effective alternative method for extracting NA from ST.

## Data availability statement

The original contributions presented in the study are included in the article/Supplementary material, further inquiries can be directed to the corresponding authors.

## Author contributions

EM: conceptualization, methodology, formal analysis, investigation, visualization, and writing—original draft. HK and NQ: formal analysis, methodology, review, and editing. BN, KM, and LK: formal analysis, investigation, review, and editing. HB and AH: formal analysis and investigation. YK: conceptualization, methodology, supervision, investigation, visualization, writing—review and editing, project administration, and funding acquisition. All authors have read and agreed to the published version of the manuscript.

## Funding

This work was supported by the OCP Phosboucraâ Foundation, Laâyoune, Morocco. Grant number: FPB_SPA001_2020.

## Conflict of interest

The authors declare that the research was conducted in the absence of any commercial or financial relationships that could be construed as a potential conflict of interest.

## Publisher’s note

All claims expressed in this article are solely those of the authors and do not necessarily represent those of their affiliated organizations, or those of the publisher, the editors and the reviewers. Any product that may be evaluated in this article, or claim that may be made by its manufacturer, is not guaranteed or endorsed by the publisher.

## Supplementary material

The Supplementary material for this article can be found online at: https://www.frontiersin.org/articles/10.3389/fnut.2023.1193509/full#supplementary-material
