# Supplementary material for: Deep eutectic solvent-ultrasound assisted extraction as a green approach for enhanced extraction of naringenin from Searsia tripartita and retained their bioactivities
Source: Front Nutr. 2023 Jun 19;10:1193509. doi: 10.3389/fnut.2023.1193509 (PMC10315493; doi:10.3389/fnut.2023.1193509)
Supplement: Supplementary file 1 [file Data_Sheet_1.docx]

Supplementary Material

Article Title

Ezzouhra El Maaiden*, Houda El Kahia, Boubker Nasser, Khadija Moustaid, Nagib Qarah, Hassan Boukcim, Abdelaziz Hirich, Lamfeddal Kouisni, Youssef El Kharrassi*

*** Correspondence:** [ezzouhra.elmaaiden@um6p.ma](mailto:ezzouhra.elmaaiden@um6p.ma) ; [youssef.elkharrassi@um6p.ma](mailto:youssef.elkharrassi@um6p.ma)

**TABLE S1.** Parameters of ultrasonic assisted extraction

| HBA/HBD Molar ratio | **Ultrasound time (min)** | **Temperature (°C)** | **Solid-to-liquid ratio (g/ml)** | **Ultrasound power (W)** | **Water content in DES %** |
| --- | --- | --- | --- | --- | --- |
| 1:1 | 5 | 30 | 1/20 | 30 | 20 |
| 1.5:1 | 10 | 40 | 1/30 | 60 | 30 |
| 2:1 | 15 | 50 | 1/40 | 75 | 40 |
| 2.5:1 | 20 | 60 | 1/50 | 90 | 50 |
| 3:1 | 25 | 70 | 1/60 | 115 | 60 |
| 3.5:1 | 30 | 80 | 1/70 | 130 | 70 |
|  | 35 |  |  | 150 |  |
|  | 60 |  |  | 180 |  |
|  | 80 |  |  |  |  |
|  | 100 |  |  |  |  |

**TABLE S2.** Independent variables and levels used for Box-Behnken design (BBD)

| Factors | Label | Levels | | |
| --- | --- | --- | --- | --- |
|  |  | ‒1 | 0 | 1 |
| Time (min) | X1 | 10 | 20 | 30 |
| Temperature (°C) | X2 | 50 | 60 | 70 |
| Solvent/Solid ratio (ml/g) | X3 | 50 | 60 | 70 |
| Ultrasound power (W) | X4 | 60 | 75 | 90 |
| Water concentration (%) | X5 | 50 | 60 | 70 |

**TABLE S3.** Box-Behnken design (BBD) matrix and response values for the NA yields

| Independent variables | | | | | | |
| --- | --- | --- | --- | --- | --- | --- |
| Run | **X1 (min)** | **X2 (°C)** | **X3 (mL/g)** | **X4(W)** | **X5 (%)** | **Y2 (ug/g)** |
| 1 | 10 (-1) | 50 (-1) | 60 (0) | 75 (0) | 60 (0) | 1244.66±3.44 |
| 2 | 20 (0) | 50 (-1) | 60 (0) | 75 (0) | 70 (1) | 1152.89±2.45 |
| 3 | 20 (0) | 50 (-1) | 50 (-1) | 75 (0) | 60 (0) | 1039.89±1.67 |
| 4 | 30 (1) | 60 (0) | 60 (0) | 60 (-1) | 60 (0) | 950.55±1.71 |
| 5 | 20 (0) | 60 (0) | 60 (0) | 75 (0) | 60 (0) | 1048.23±2.67 |
| 6 | 20 (0) | 60 (0) | 70 (1) | 60 (-1) | 60 (0) | 1068.88±2.78 |
| 7 | 20 (0) | 60 (0) | 60 (0) | 75 (0) | 60 (0) | 1054.50±1.87 |
| 8 | 10 (-1) | 60 (0) | 60 (0) | 75 (0) | 50 (-1) | 1130.23±1.45 |
| 9 | 10 (-1) | 60 (0) | 60 (0) | 90 (1) | 60 (0) | 1151.56±2.09 |
| 10 | 30 (1) | 50 (-1) | 60 (0) | 75 (0) | 60 (0) | 911.65±1.54 |
| 11 | 20 (0) | 70 (1) | 60 (0) | 60 (-1) | 60 (0) | 932.12±1.34 |
| 12 | 20 (0) | 60 (0) | 70 (1) | 75 (0) | 70 (1) | 1173.23±1.98 |
| 13 | 30 (1) | 60 (0) | 60 (0) | 75 (0) | 70 (1) | 994.12±1.77 |
| 14 | 20 (0) | 60 (0) | 50 (-1) | 90 (1) | 60 (0) | 1067.34±0.89 |
| 15 | 20 (0) | 60 (0) | 50 (-1) | 60 (-1) | 60 (0) | 1055.00±1.82 |
| 16 | 20 (0) | 70 (1) | 71 (1) | 75 (0) | 60 (0) | 945.79±1.11 |
| 17 | 30 (1) | 70 (1) | 60 (0) | 75 (0) | 60 (0) | 885.68±1.03 |
| 18 | 20 (0) | 60 (0) | 60 (0) | 90 (1) | 50 (-1) | 1031.96±2.01 |
| 19 | 20 (0) | 60 (0) | 60 (0) | 75 (0) | 60 (0) | 1049.99±2.17 |
| 20 | 20 (0) | 60 (0) | 60 (0) | 90 (1) | 70 (1) | 1110.11±2.33 |
| 21 | 10 (-1) | 70 (1) | 60 (0) | 75 (0) | 60 (0) | 972.84±1.34 |
| 22 | 10 (-1) | 60 (0) | 60 (0) | 60 (-1) | 60 (0) | 1198.27±1.89 |
| 23 | 20 (0) | 70 (1) | 60 (0) | 90 (1) | 60 (0) | 925.76±1.34 |
| 24 | 30 (1) | 60 (0) | 60 (0) | 75 (0) | 50 (-1) | 979.86±1.21 |
| 25 | 20 (0) | 60 (0) | 70 (1) | 90 (1) | 60 (0) | 1077.04±2.10 |
| 26 | 20 (0) | 60 (0) | 50 (-1) | 75 (0) | 70 (1) | 1137.16±2.21 |
| 27 | 20 (0) | 70 (1) | 50 (-1) | 75 (0) | 60 (0) | 941.79±1.89 |
| 28 | 10 (-1) | 60 (0) | 60 (0) | 75 (0) | 70 (1) | 1205.29±2.91 |
| 29 | 30 (1) | 60 (0) | 70 (1) | 75 (0) | 60 (0) | 994.89±1.03 |
| 30 | 20 (0) | 60 (0) | 70 (1) | 75 (0) | 50 (-1) | 1064.02±1.67 |
| 31 | 20 (0) | 60 (0) | 60 (0) | 75 (0) | 60 (0) | 1036.97±1.33 |
| 32 | 20 (0) | 50 (-1) | 60 (0) | 90 (1) | 60 (0) | 1098.08±1.56 |
| 33 | 10 (-1) | 60 (0) | 70 (1) | 75 (0) | 60 (0) | 1203.28±2.88 |
| 34 | 20 (0) | 50 (-1) | 60 (0) | 60 (-1) | 60 (0) | 1012.92±1.43 |
| 35 | 20 (0) | 60 (0) | 60 (0) | 60 (-1) | 70 (1) | 1118.12±1.76 |
| 36 | 10 (-1) | 60 (0) | 50 (-1) | 75 (0) | 60 (0) | 1193.26±1.69 |
| 37 | 20 (0) | 50 (-1) | 60 (0) | 75 (0) | 50 (-1) | 1024.94±1.53 |
| 38 | 30 (1) | 60 (0) | 60 (0) | 90 (1) | 60 (0) | 982.86±1.12 |
| 39 | 20 (0) | 70 (1) | 60 (0) | 75 (0) | 50 (-1) | 934.77±1.09 |
| 40 | 30 (1) | 60 (0) | 50 (-1) | 75 (0) | 60 (0) | 968.84±2.12 |
| 41 | 20 (0) | 50 (-1) | 70 (1) | 75 (0) | 60 (0) | 1081.05±2.84 |
| 42 | 20 (0) | 70 (1) | 60 (0) | 75 (0) | 70 (1) | 958.82±1.70 |
| 43 | 20 (0) | 60 (0) | 60 (0) | 75 (0) | 60 (0) | 1041.98±2.32 |
| 44 | 20 (0) | 60 (0) | 60 (0) | 75 (0) | 60 (0) | 1026.95±3.01 |
| 45 | 20 (0) | 60 (0) | 50 (-1) | 75 (0) | 50 (-1) | 1075.04±1.78 |
| 46 | 20 (0) | 60 (0) | 60 (0) | 60 (-1) | 50 (-1) | 1039.97±2.37 |
